# Supplementary material for: A fault-intrusion-tolerant system and deadline-aware algorithm for scheduling scientific workflow in the cloud
Source: PeerJ Comput Sci. 2021 Nov 2;7:e747. doi: 10.7717/peerj-cs.747 (PMC8576554; doi:10.7717/peerj-cs.747)
Supplement: Supplemental Information 6 [file peerj-cs-07-747-s006.zip › Myproject1.1/docs/org/cloudbus/cloudsim/lists/package-summary.html]

org.cloudbus.cloudsim.lists


JavaScript is disabled on your browser.


- Overview
- Package
- Class
- Use
- Tree
- Deprecated
- Index
- Help

- Prev Package
- Next Package

- Frames
- No Frames

- All Classes

# Package org.cloudbus.cloudsim.lists

- Class Summary

  | Class | Description |
  | --- | --- |
  |  |  |
  | --- | --- |
  | CloudletList | CloudletList is a collection of operations on lists of Cloudlets. |
  | HostList | HostList is a collection of operations on lists of hosts. |
  | PeList | PeList is a collection of operations on lists of PEs. |
  | ResCloudletList | ResCloudletList is a collection of operations on lists of ResCloudlets. |
  | VmList | VmList is a collection of operations on lists of VMs. |

- Overview
- Package
- Class
- Use
- Tree
- Deprecated
- Index
- Help

- Prev Package
- Next Package

- Frames
- No Frames

- All Classes
